# Interest in mHealth Among Patients With Low Back Pain: Cross-Sectional Study

**DOI:** 10.2196/48729

**Published:** 2024-02-12

**Authors:** Jonas Ammundsen Ipsen, Louise Fleng Sandal, Natalie Hong Siu Chang, Berit Schiøttz-Christensen, Karen Søgaard, Anders Hansen

**Affiliations:** 1 Department of Regional Health Research University of Southern Denmark Odense Denmark; 2 Department of Physical Therapy and Occupational Therapy Lillebaelt Hospital University Hospital of Southern Denmark Kolding Denmark; 3 Department of Sports Science and Clinical Biomechanics University of Southern Denmark Odense Denmark; 4 Medical Research Spine Centre of Southern Denmark University Hospital of Southern Denmark Middelfart Denmark; 5 Research Unit of General Practice University of Southern Denmark Odense Denmark; 6 Department of Clinical Research University of Southern Denmark Odense Denmark

**Keywords:** low back pain, mHealth solutions, mobile health, characteristics, patient interest, transferability, representativeness

## Abstract

**Background:**

Digitally supported self-management tailored to an individual’s need, in addition to usual care, may reduce pain-related disability compared to usual care alone, and patients with low back pain (LBP) using mobile health (mHealth) solutions express positive experiences. Hence, implementing mHealth solutions designed to support self-management is desirable from a clinical and patient perspective. Easily accessible mHealth solutions that can support the self-management of patients with LBP are available, but interest may be subgroup specific. Understanding the characteristics and preferences of patients with LBP labeled as interested may help to reach relevant LBP patient groups and inform the development and implementation of effective interventions with mHealth for patients with LBP.

**Objective:**

This study aims to explore the proportion of patients with LBP labeled as interested in testing an mHealth solution designed to support self-management in addition to usual care and to assess how these patients differ from those who were labeled as not interested.

**Methods:**

This exploratory cross-sectional study analyzed demographic and patient-reported outcomes from the SpineData registry, a Danish registry of patients with LBP in an outpatient setting. Between February and December 2019, the SpineData registry was used to assess the preliminary eligibility of patients for a clinical trial (selfBACK). Patients were labeled as interested or uninterested depending on if they responded to an invitation to be tested for eligibility for the trial Outcomes were selected from the International Classification of Functioning core set of LBP using a clinical approach. Associations were assessed in a backward selection process, and the proportion of variance explained was assessed with pseudo-*R*^2^ statistic.

**Results:**

This study included 843 patients, with 181 (21%) individuals labeled as interested in participating in the selfBACK trial. Notably, the cohort labeled as interested differed from their uninterested counterparts in two key aspects: age (36-65 years: 116/181, 64.1% vs 347/662, 52.4%; *P*=.003) and smoking status (smokers: 22/181, 12.5% vs 174/662, 26.6%; *P*<.001). Those aged 36-65 years had higher odds of being labeled as interested compared to individuals aged 18-35 years (odds ratio [OR] 0.43, 95% CI 0.26-0.71) and those 65 years or older (OR 0.77, 95% CI 0.53-1.15). Nevertheless, age accounted for only a modest proportion of variance (*R*^2^=0.014). Smokers demonstrated lower odds of being labeled as interested (OR 0.39, 95% CI 0.24-0.64), with smoking status explaining a similarly small proportion of variance (*R*^2^=0.019). Collectively, age and smoking status accounted for 3.3% of the variance.

**Conclusions:**

Our investigation revealed that 181 (21%) individuals with LBP invited to participate in the mHealth solution trial for self-management expressed interest. Generally, the characteristics of those labeled as interested and uninterested were comparable. Of note, patients aged 36-65 years had a higher frequency of being labeled as interested compared to their younger and older counterparts.

## Introduction

Digital health interventions delivered with smartphones (mobile health [mHealth] solutions) are accessible to most patients across age, geography, and socioeconomic status. Thus, clinicians’ expectations of mHealth solutions are significant, and the availability of new solutions on the commercial market every day also indicates a strong general interest in using mHealth solutions [1,2]. Nevertheless, many mHealth solutions have limited download rates, and if downloaded, the use can be scarce [3,4]. This discrepancy may indicate a need for a better understanding of potential users and their characteristics.

For patients with low back pain (LBP; not attributable to a recognizable, known specific pathology such as infections, fractures, or structural deformity), self-management support is recommended as the first line of treatment [5-8]. This may involve empowering patients to know when to consult for diagnostic assessment, symptom relief, or advice [9]. Digitally supported self-management may be delivered through smartphone apps or digital platforms to facilitate and enhance such self-management practices. Research indicates that the integration of such digitally supported self-management strategies, when combined with standard care, can lead to a reduction in pain-related disability [10]. Further, evidence supports that mHealth solutions designed to support self-management are accepted by patients with chronic LBP [9]. Therefore, there is a growing interest in implementing mHealth solutions designed to support self-management from both clinical and patient perspectives. However, despite the potential benefits, the level of acceptance and use of these interventions remains an area that requires further investigation.

However, studies on other patient groups using mHealth solutions report that lower age, higher education, higher income, higher BMI, and higher self-perceived health are associated with increased use [4,11]. In contrast, the cost of using these apps is a significant barrier [11].

Individuals with LBP who use mHealth solutions to self-manage may thus represent a specific subset within the general population. Therefore, this study aimed to investigate the percentage who expressed interest in participating in a trial evaluating an mHealth solution designed for self-management alongside standard care, as well as to evaluate potential distinctions between those who were labeled as expressing interest and those who were not.

## Methods

### Study Design

This exploratory cross-sectional study used demographic and patient-reported outcomes (PROs) from an internet-based multiuser clinical registry (SpineData) [12]. Reporting follows the STROBE (Strengthening the Reporting of Observational Studies in Epidemiology) guidelines [13].

### Setting

Data were collected at the Spine Centre of Southern Denmark, an outpatient hospital that performs clinical spine evaluations [12]. General practitioners or chiropractors typically refer patients to the Spine Centre, which performs a multidisciplinary assessment of its patients, with more than 10,000 new cases yearly.

Before patients are evaluated at the Spine Centre, they provide information in the local SpineData registry [14]. The registry is designed based on the biopsychosocial model of health, and information is collected across the health domains of pain, activity limitation, work participation, psychological factors, physical impairment, and contextual factors [12]. To mitigate nonresponse and missing information, SpineData uses a “waterfall” model (eg, patients in employment are not asked to respond to causes for unemployment). SpineData has an overall completion rate of 80% and approximately 60% of patients agreed to their responses being used for research [14]. The use of this registry allows for the comprehensive assessment of patients consulted at the Spine Centre and provides a rich source of data for research studies, such as the one presented in this paper.

### Participants

Between February and December 2019, SpineData was used to identify eligible patients based on the following criteria: consenting to be contacted for research projects, proficiency in Danish, and experiencing LBP in the past 14 days that exceeded their leg pain in severity. Patients with previous back surgery, who were actively filing for a pension, or who were younger than 18 years were not invited. All patients matching the eligibility criteria were sent a letter of invitation to hear more about the selfBACK trial. One reminder was sent. The patients who did not respond to either invitation or reminder were labeled uninterested. The selfBACK trial investigated the effectiveness of the selfBACK digital decision support system that provided patients with LBP individually tailored digital support in an app format using three content domains: (1) physical activity, (2) education, and (3) exercise programs. The trial investigated the additive effect of the selfBACK system in addition to usual care. Participants in this trial were recruited from primary health care such as chiropractors, physiotherapists, and general practitioners in addition to the Spine Center of Southern Denmark. Recruitment was performed in Denmark and Norway. The population within this study concerns the pool of patients seen at the Spine Center, who would have received an invitation to eligibility screening to the selfBACK trial based on their answers given in the SpineData clinical registry. In this study, all patients who matched the preliminary eligibility criteria for the selfBACK trial were included [15].

### Outcomes

The variables of interest were selected from the SpineData registry, based on the International Classification of Function core set for LBP and clinical reasoning [16]. The demographics and clinical characteristics comprised the domains of pain, activity limitation, work participation, and psychological and contextual factors (Textbox 1).

**Detailed description of the content and handling of included outcomes.**

**Sex**

- Male or female

**Age**

- Patients were categorized into age groups ≤35, 36-65, and >65 years.

**BMI**

- The anthropometric variables of height and weight were used to calculate BMI (kg/m^2^).

**Smoker status**

- Categorical variable that was dichotomized to smoker and nonsmoker strata. If a patient indicated cigarette use of any kind, they were categorized as a smoker.

**Alcohol consumption**

- Categorical variable that was stratified into two groups based on the consumption of more than 14 alcoholic beverages a week. The threshold was based on the recommendation of the Danish Ministry of Health [17].

**Comorbidities**

- This variable was based on four dichotomous variables: allergies, including medication; cancers; heart disease; and lung disease. If a patient replied yes to one of these variables, they were categorized as having comorbidities.

**Current work status**

- The work status variables consisted of different ways of participating in the labor market: working full- or part-time, flex job, in education, job training due to inability to maintain habitual job function, unemployed, early retirement, pensions, stay at home, and other. The variable was dichotomized to working or not by grouping patients indicating working part- or full-time, flex, and students in one group and the remainder in another group.

**Multiple pain sites**

- SpineData contains a freehanded pain drawing. The pain drawing was post defined into 46 anatomical regions. In this study, the regions were grouped into 9 areas: neck, shoulders, upper back, elbows, lower back, wrists/hands, hips/thighs, knees, and ankles/feet, inspired by Øverås et al [18]. Patients with two or more pain sites were considered as having multiple pain sites.

**Average back pain**

- The average back pain in the last 14 days was measured on a 0-10 numeric rating scale, with 10 indicating the worst imaginable pain.

**STarT BaCK screening tool [19]**

- The STarT Back scores categorize patients into three strata based on their risk of developing chronicity: low risk, moderate risk, and high risk of chronicity:
  Low risk: <3
  Moderate risk: ≥4 and subscore ≤3
  High risk: ≥4 and subscore ≥4

**EQ-5D-5L-VAS [20]**

- Numeric rating scale score spanning from 0 to 100, with 100 representing the best possible health state

**Oswestry Disability Index (ODI) [21]**

- The ODI is a questionnaire containing 10 items that are scored from 0 to 5. The maximum score is 50 points, which indicates that the patient is bedbound. The ODI has been found valid for patients with low back pain [22].
- To estimate the patients’ functional level, the ODI Stata package was used. The ODI package allows for the imputation of data for one missing value. The missing values in one section were replaced with the average score for all sections.

**Anxiety [23]**

- Numeric score rating from 0 to 10, with 0 indicating no anxiety and 10 a high degree of anxiety

**Social isolation [23]**

- Numeric score rating from 0 to 10, with 0 indicating no loneliness and 10 a high degree of loneliness

**Catastrophization (terrible pain that will never improve) [23]**

- Numeric score rating from 0 to 10, with 0 indicating no catastrophization and 10 a high degree of catastrophization

**Catastrophization (the pain is overwhelming) [23]**

- Numeric score rating from 0 to 10, with 0 indicating no catastrophization and 10 a high degree of catastrophization

**Risk of persisting pain [23]**

- Numeric score rating from 0 to 10, with 0 indicating no risk of persisting pain and 10 a high risk of persisting pain

**Feelings of sadness, depression, or hopelessness [23]**

- Numeric score rating from 0 to 10, with 0 indicating no feelings of depression and 10 a constant presence of depression

**Loss of interest or joy [23]**

- Numeric score rating from 0 to 10, with 0 indicating no loss of interest or joy and 10 never feeling interest or joy

**Fearing activity will damage the back [23]**

- Numeric score rating from 0 to 10, with 0 indicating no fear that physical activity will damage the back and 10 completely agreeing that physical activity will damage the back

**Fearing activity will increase the pain [23]**

- Numeric score rating from 0 to 10, with 0 indicating completely disagreeing to avoid physical activity and 10 completely agreeing to avoid physical activity

### Exposure

Patients were allocated into two groups based on their response to being invited for eligibility screening for the selfBACK trial. Those who responded positively to the invitation to be screened were labeled as interested in using the mHealth solution, whereas those who did not respond were labeled as uninterested.

### Statistical Methods

The demographics and baseline characteristics of patients who were or were not labeled as interested in the digital mHealth intervention were assessed using the *χ*^2^ test for categorical variables and 2-tailed Student *t* test for continuous variables. Baseline characteristics are reported as the proportion and percentage or mean and SD.

To assess the strength of associations between PROs and patients labeled as interested in mHealth or not, we used univariate and multivariate logistic regression analysis with an odds ratio (OR) and 95% CI. The associations were assessed in a backward selection process, and the proportion of variance explained was assessed with McFadden pseudo-*R*^2^ statistic. Statistical analyses were performed with Stata statistical software (Release 17; StataCorp LLC). Missing information was handled using pairwise deletion. The ODI Stata package allows for data imputation for one missing value. The missing values in one section were replaced with the average score for all sections. To avoid overparameterizing the model, we aimed for a 1:10 patient-to-variable ratio.

### Ethical Considerations

The Region of Southern Denmark was the data controller for this project, which is included in its records on personal data processing activities (file 21/13433). Data processing in the project was regulated by the Danish Act on Research Ethics Review of Health Research Projects section 14, subsection 2, which states that health research based solely on questionnaire surveys and registry data is exempt from the obligation to notify the committees. Following the Danish Health Care Act, we obtained approval for using hospital record data for scientific purposes from the council of the Region of Southern Denmark (file 21/25588). After merging, analyses were run on pseudonymized data, and the results presented in this manuscript do not enable the identification of single data participants. Hence, following national laws, no additional informed consent was collected and no remuneration was offered to patients.

## Results

### Overview

From February to the end of December 2019, 5796 patients (~80% of those invited) completed the SpineData registry before their diagnostic assessment at the Spine Centre. Of the total sample, 843 (15%) were invited to the selfBACK trial. The mean age of the cohort was 52 (SD 16.2) years, with an even distribution of sexes (male: n=429, 50.1%), and a mean BMI of 27.5 kg/m^2^.

Of the 843 patients invited to the eligibility screen for the trial, 181 (21%) accepted the invitation and were stratified into the group who were labeled as interested in the mHealth solution.

Of the 21 included variables, 8 had complete responses, and none of the remaining 13 variables had more than 2.5% missing responses.

### Comparison of Patients Who Were Labeled as Interested and Uninterested in an mHealth Solution

Patients labeled as interested in using the mHealth solution were aged 36-65 years (*P*=.003) and had a lower proportion of smokers (*P*<.001) compared to the patients labeled as uninterested. The remaining variables were not different between the patients labeled as interested and uninterested (Table 1).

**Table 1:** Baseline characteristics of patients labeled as interested in the mobile health solution compared to the uninterested patients.

Baseline characteristic^a^		Interested (n=181)	Uninterested (n=662)	*P* value
Female, n (%)		93 (51.3)	321 (48.5)	.49
**Age (years), n (%)**				.003
	18-35	21 (11.6)	146 (22.1)	
	36-65	116 (64.1)	347 (52.4)	
	>65	44 (24.3)	169 (25.5)	
BMI (kg/m^2^), mean (SD)		28.1 (5.8)	27.2 (5.0)	.05
Smokers, n (%)		22 (12.5)	174 (26.6)	<.001
<14 alcohol consumption per week, n (%)		173 (95.5)	636 (96.0)	.76
Has comorbidities, n (%)		84 (46.7)	208 (42.3)	.29
Working, n (%)		95 (52.4)	367 (55.4)	.49
Has multiple pain sites, n (%)		136 (75.1)	462 (69.7)	.24
Average back pain (score range: 0-10), mean (SD)		6.3 (2.0)	6.3 (1.9)	.91
**STarT Back tool, n (%)**				.34
	Low risk	54 (29.8)	164 (24.7)	
	Moderate risk	46 (25.5)	169 (25.5)	
	High risk	81 (44.7)	329 (49.7)	
EQ-5D-5L-VAS (score range: 0-100), mean (SD)		59.0 (22.1)	55.2 (23.0)	.05
Oswestry Disability Index (score range: 0-50), mean (SD)		30.3 (15.6)	31.1 (14.9)	.50
Anxiety (score range: 0-10), mean (SD)		3.8 (3.0)	3.8 (3.1)	.99
Loneliness (score range: 0-10), mean (SD)		1.4 (2.4)	1.3 (2.2)	.67
Catastrophization (terrible pain that will never improve; score range: 0-10), mean (SD)		4.8 (2.9)	5.0 (3.0)	.48
Catastrophization (the pain is overwhelming; score range: 0-10), mean (SD)		3.7 (3.1)	4.1 (3.1)	.24
Risk of persisting pain (score range: 0-10), mean (SD)		6.8 (2.6)	6.8 (2.6)	.91
Sadness (score range: 0-10), mean (SD)		3.5 (3.1)	3.6 (3.1)	.71
Loss of interest or joy (score range: 0-10), mean (SD)		4.3 (3.3)	4.3 (3.2)	.88
Fearing activity will damage the back (score range: 0-10), mean (SD)		3.4 (2.9)	3.8 (3.2)	.08
Fearing activity will increase the pain (score range: 0-10), mean (SD)		4.8 (3.2)	4.4 (3.3)	.16

^a^Missing: 8 of the 21 variables had complete responses, and none of the remaining 13 variables had more than 2.5% missing responses.

Our results suggest that patients aged 36-65 years were more likely to be labeled as interested in mHealth solutions compared to patients between 18-35 years (OR 0.43, 95% CI 0.026-0.711) and 65 years or older (OR 0.77, 95% CI 0.525-1.153) and explained a limited proportion of variance (*R*^2^=0.014)*.* Smoker (OR 0.39, 95% CI 0.244-0.636) and the association explained a limited proportion of variance (*R*^2^=0.019). Combined, the associations of age and smoking explained 3.3% of the proportion of variance.

These findings were supported by univariate regression analysis and a comparison of patients who were labeled as expressing interest in the mHealth solution to those who did not. The proportion of variance explained in the group of patients labeled as interested in mHealth solutions across the 21 selected variables was 0.059, with age and smoking status accounting for 0.033 of the variance (Table 2).

BMI (*P*=.05), overall perception of health measured using the EQ-5D-5L-VAS score (*P*=.05), and fear that activity will damage the back (*P*=.08) were borderline significant.

**Table 2:** Associations to being labeled as interested and proportion of variance explained.

		Odds ratio (95% CI)	SE	*Z*	People invited, N		*P* value (*P*>|z|)		*R* ^2^	
**Age (years)**						830				0.014
	36-65 (reference)	—	—	—			—			
	18-35	0.43 (0.02-0.71)	0.114	–3.17			.002			
	>65	0.77 (0.52-1.15)	0.151	–1.48			.14			
**Smoking**						830				0.019
	Nonsmoker (reference)	—	—	—	—		—		—	
	Smoker	0.39 (0.24-0.64)	0.098	–3.72			<.001			

## Discussion

### Principal Results

This study aimed to explore the proportion of patients with LBP who were labeled as interested in using an mHealth solution designed to support self-management in addition to usual care and assess how these patients differed from those who were labeled as not interested. We found that 21% of the eligible patients were labeled as interested in using the mHealth solution. The groups had no statistically significant differences except that patients labeled as interested were more frequently within the 36-65 years age range and were nonsmokers.

### Comparison With Prior Work

Previous evidence of the characteristics and associations of patients with LBP and their interest in mHealth solutions is limited. Contrary to Krebs and Duncan [4], we found a nonsignificant association between BMI and no association between being younger and labeled as interested in mHealth solutions. The key differences between Krebs and Duncan [4] and this study are the target populations (general population) and the type of mHealth solutions included (fitness apps or calorie trackers). Similar to our results, Philip et al [24] identified an association between higher age and increased use of mHealth solutions among patients with chronic pain. We suggest that the differences in results between Krebs and Duncan [4], Philip et al [24], and this study were due to differences between participants from the general population and patients with LBP or chronic pain. Three recent studies have assessed the characteristics and associations of users and nonusers of different mHealth apps, all using participants from the general population, but still lacking consensus. Walrave et al [25] identified no sociodemographic differences between users and nonusers of contact tracking alert apps, including the Belgian Corona alert app. A study of the general US population identified strong associations of age, gender, and education level with the use of fitness apps and calorie counters [26]. Lim et al [27] identified that female patients with higher education were more prevalent users of mHealth apps. Although this lack of consensus regarding patient interest could indicate a call for more research, it could also reflect that the interest in mHealth solutions may be characterized by patients’ preferences and perspectives on the relevance of mHealth solutions.

### Strengths and Limitations

This study benefitted from several strengths. First, we had access to comprehensive information on the patients participating through the SpineData registry. Further, we benefitted from the fact that SpineData has been in routine use for several years and is frequently updated per clinician and evidence demand [14]. Thus, the PROs were collected using validated questionnaires or questions designed for the LBP population and International Classification of Function core set [14,16,28,29]. The included patients were identified using a computer algorithm, and patients were sent one invitation and one reminder invitation to be screened for eligibility. Thus, the risk of unconscious bias in the recruitment was eliminated. However, using a single data source (SpineData) also limited the variables available to investigate in the univariate model. Low education and economic status have been associated with limited use and adoption of mHealth solutions [26,30], but this information was unavailable in SpineData. Smoking is reportedly more prevalent among patients with a lower socioeconomic or sociodemographic status [31,32]. Further, the use of one registry meant we only had access to PROs, which may be affected by recall bias. The statistically significant difference between being labeled as interested in mHealth solutions by smoking status could reflect a difference in education level. Thus, education level is a parameter that could differentiate the patients labeled as interested and those labeled as uninterested in the mHealth solution, although this hypothesis remains unanswered. Patients referred to the Spine Centre usually have pain for extended periods and at a higher intensity than patients in the primary sector [33]. Thus, these patients potentially have more complex LBP issues than those with LBP who were not referred, which means that our study population may be a subgroup of the general LBP population. The terms “interested” and “uninterested” pose a challenge due to their vague nature. We recognize the distinction between demonstrating a “cursory” interest and moving toward actual participation. After extensive discussions among authors, we chose the terms “interested” and “uninterested.” Despite their less-than-optimal nature, we believe these terms best suit the context where we categorize patients based on their response to an invitation, progressing from screening to eligibility for participation in a trial evaluating an mHealth solution supporting self-management in patients with LBP. Further, some patients might be interested in testing an mHealth solution but uninterested in participating in a trial or vice versa. Further, those labeled as uninterested in the mHealth solution in this study might see advantages in mHealth solutions that they found more relevant like how to stop smoking or lose weight [34]. This study only addresses patient characteristics; however, investigating clinicians’ perspectives on the use and adoption of mHealth solutions in LBP self-management will similarly inform on barriers to and facilitators of increased mHealth adoption in clinical practice. However, as the SpineData clinical registry only entails patient data, this perspective was not possible in this study. Thus, the results of this study should be interpreted with caution regarding generalizability, and future qualitative or mixed methods studies could explore patients’ preferences and perceptions of the relevance of mHealth solutions. Another important area of research can be clinicians’ acceptability of mHealth solutions and the need for rigorous demonstrations of safety and efficacy to alleviate any reservations or hesitance among clinicians.

### Conclusion

This study aimed to explore the characteristics of patients labeled as interested or uninterested in participating in a trial testing an mHealth solution designed to improve self-management. Our study identified that 21% (n=181) of eligible patients with LBP were labeled as interested in participating in the trial testing an mHealth solution to support self-management. Overall, the patients labeled as interested and uninterested, except for age and smoking status were similar. Interestingly, patients aged 36-65 years were more frequently labeled as interested in the mHealth solution. Thus, patients aged 36-65 years may be more interested in adopting mHealth solutions. How to increase interest in mHealth solutions among younger and older patients with LBP is an important consideration for future research and developers, especially as the findings of the selfBACK trial indicate an increased effect for older patients.

## Abbreviations

LBP: low back pain
mHealth: mobile health
OR: odds ratio
PRO: patient-reported outcome
STROBE: Strengthening the Reporting of Observational Studies in Epidemiology
